# Inorganic carbon physiology underpins macroalgal responses to elevated CO_2_

**DOI:** 10.1038/srep46297

**Published:** 2017-04-18

**Authors:** Christopher E. Cornwall, Andrew T. Revill, Jason M. Hall-Spencer, Marco Milazzo, John A. Raven, Catriona L. Hurd

**Affiliations:** 1 Institute for Marine and Antarctic Studies, University of Tasmania, Hobart, Tasmania 7001, Australia; 2 School of Earth Sciences, Oceans Institute, and ARC Centre of Excellence for Coral Reef Studies, University of Western Australia, Crawley, Western Australia 6009, Australia; 3 CSIRO Oceans and Atmosphere, Hobart, Tasmania 7000, Australia; 4 Marine Biology and Ecology Research Centre, Plymouth University, Plymouth, UK; 5 Shimoda Marine Research Centre, University of Tsukuba, Japan; 6 DiSTeM, CoNISMa, University of Palermo, Palermo, Italy; 7 Division of Plant Science, University of Dundee at the James Hutton Institute, Invergowie, Dundee, DD2 5DA, UK; 8 School of Plant Biology, University of Western Australia, Crawley, Western Australia 6009, Australia

## Abstract

Beneficial effects of CO_2_ on photosynthetic organisms will be a key driver of ecosystem change under ocean acidification. Predicting the responses of macroalgal species to ocean acidification is complex, but we demonstrate that the response of assemblages to elevated CO_2_ are correlated with inorganic carbon physiology. We assessed abundance patterns and a proxy for CO_2_:HCO_3_^−^ use (δ^13^C values) of macroalgae along a gradient of CO_2_ at a volcanic seep, and examined how shifts in species abundance at other Mediterranean seeps are related to macroalgal inorganic carbon physiology. Five macroalgal species capable of using both HCO_3_^−^ and CO_2_ had greater CO_2_ use as concentrations increased. These species (and one unable to use HCO_3_^−^) increased in abundance with elevated CO_2_ whereas obligate calcifying species, and non-calcareous macroalgae whose CO_2_ use did not increase consistently with concentration, declined in abundance. Physiological groupings provide a mechanistic understanding that will aid us in determining which species will benefit from ocean acidification and why.

Ocean acidification is caused by seawater absorption of anthropogenically-derived CO_2_^1^, and is a major threat to many marine ecosystems through alterations in organism physiology and changes in ecological interactions^2^. Ocean acidification will impact organisms via increased dissolution rates of calcareous species^3^,^4^, altered behaviour of marine invertebrates and fish^2^, and by increasing the supply of dissolved inorganic carbon (DIC) to some autotrophs^5^,^6^,^7^. These effects will be particularly strong at an ecosystem level if they affect either foundation or keystone species^2^,^8^,^9^. Macroalgae provide food and habitat to tens of thousands of species in shallow-water regions throughout the world^10^,^11^, and all utilise external CO_2_ and/or bicarbonate (HCO_3_^−^) for photosynthesis, with CO_2_ being the ultimate substrate for the enzyme RuBisCO^12^,^13^ which fixes inorganic carbon. Calcareous macroalgae can be directly impacted by ocean acidification due to increased dissolution and reduced calcification rates^7^,^14^,^15^,^16^. Indirectly, calcareous macroalgae face increased competition from non-calcareous macroalgae (hereafter “fleshy macroalgae”) that could benefit from elevated CO_2_^17^,^18^,^19^. Coralline algae are predicted to be at particular risk from ocean acidification^14^,^20^,^21^; they provide substrata for the settlement of marine invertebrates and also create and bind together reefs in shallow waters from the poles to the tropics^22^. However, while the negative response of obligate calcifying macroalgae to ocean acidification is well established^3^,^23^, there are still large gaps in our knowledge regarding general patterns of how fleshy macroalgae will respond to ocean acidification, making predictions of future ecological dynamics difficult.

Physiological mechanisms underpinning the responses of fleshy macroalgae to ocean acidification are poorly understood^5^, although they are likely related to methods of DIC uptake^18^, summarised in Fig. 1. Carbon dioxide concentrating mechanisms (CCMs) allow continued DIC uptake when CO_2_:O_2_ ratios are high (e.g. when seawater is highly modified by photosynthesis), or when CO_2_ concentrations are insufficient to maximise the rate of photosynthesis^12^. CCMs act mostly via the active uptake of HCO_3_^−^, and there are multiple types of CCMs with varying energetic efficiencies^12^,^24^. The presence of CCMs in macroalgae suggests that ocean acidification may not benefit fleshy macroalgae, as DIC is not always limiting for growth or photosynthesis^25^. Conversely, macroalgae lacking CCMs (hereafter “non-CCM” macroalgae), or with CCMs with low affinities for DIC, could benefit from ocean acidification due to the alleviation of any CO_2_ limitation^6^,^18^. Additionally, DIC uptake via a CCM is marginally more energetically expensive than CO_2_ uptake via diffusion when the lowest costs of photorespiration (i.e. oxygenase rather than carboxylase activity with subsequent energetic waste) are included: this energetic cost of using a CCM assumes that leakage of DIC from the cell could occur because DIC concentrations within the cell, compared to seawater, increase^24^. Therefore, species could gain energetic savings from increasing CO_2_ concentrations if external seawater CO_2_ concentrations exceed that produced internally by the CCM, and replacement of the CCM with diffusive CO_2_ entry to Rubisco could occur^24^,^26^,^27^. This replacement occurs within one or a few generations in photosynthetic organisms, leading to the downregulation of the CCM and long-term energetic savings, which could manifest as benefits such as elevated growth rates, or competitive advantages due to ocean acidification^18^.

Predicting how the multifaceted physiological effects of ocean acidification will combine to influence macroalgal assemblages is difficult. This challenge is amplified by complex ecological and environmental processes that exert pressures on macroalgal species in ways that are difficult to capture in laboratory experiments. The ecological outcomes of combined positive and negative effects of elevated CO_2_ can be observed at volcanic CO_2_ seeps^28^, which provide a field setting in which to test hypotheses regarding how ocean acidification could influence multi-species assemblages. Coralline algae and calcareous green algae (*Halimeda* and *Acetabularia* spp.) decline at seeps where CO_2_ concentrations are similar to and below those predicted to occur in the ocean at the end of the century^28^. As CO_2_ concentrations rise closer to the seeps, non-calcareous algae increase in abundance, but not diversity^29^, and include assemblages comprised of diatoms, cyanobacteria, filamentous macroalgae and fucoids^28^,^30^,^31^. Many species (or groups of species) belonging to the morphological category of filamentous or turf macroalgae benefit from elevated CO_2_^32^. However, this effect is not ubiquitous^29^, particularly in tropical ecosystems^33^, and not all natural ecosystems or seep locations contain ephemeral turf species. In addition, the term turf comprises many unrelated taxa that could have different physiologies^34^, which will therefore respond in different ways to elevated CO_2_^18^,^33^. Therefore, it is presently unknown why some fleshy macroalgal species appear to benefit from elevated CO_2_ concentrations at these sites and others species do not^32^,^35^.

It has been suggested that macroalgae capable of utilising additional CO_2_ may benefit at the expense of species that are not capable of utilising increasing CO_2_^32^. While this seems elementary, determining which species will utilise additional CO_2_ is impossible without an understanding of organismal physiology. Seaweed functional form^36^ – such as the presence or absence of calcification – can determine the likely-hood of an organism responding negatively to ocean acidification. However, using functional form or morphological groups alone will not determine whether a species is capable of utilising additional CO_2_ under ocean acidification. Functional form or taxonomic groups have been inadequate in explaining the responses of terrestrial plants to climate change, and it has been suggested that assigning plants to physiological functional groups may serves as a better predictive tool^37^,^38^.

Hepburn *et al*.^18^ suggested a template for predicting the responses of the future abundance of macroalgae to elevated CO_2_ based on their inorganic carbon physiology: calcareous species will decline, non-CCM species will increase, and CCM species will either be unaffected or may increase. However, to date these predictions regarding fleshy macroalgae remain untested in the field, and the groups used by Hepburn *et al*. may even be too broad. Here, we refine this template and combine it with the predictions of Connell *et al*.^32^, who state that species capable of utilising additional CO_2_ will benefit from ocean acidification at the expense of those that cannot. For fleshy macroalgae, we group species as: (1) non-CCM species; (2) CCM species capable of utilising additional CO_2_; and (3) CCM species not capable of utilising additional CO_2_. We expand group (2) into species that have either (a) CCMs with a low affinity for DIC (carbon-limited CCM species), or (b) have CCMs that can be downregulated (i.e. lower CCM activity under elevated CO_2_ concentrations compared to ambient conditions). Group (3) contains CCM species with high affinities for CO_2_, whose CCM does not downregulate due to additional CO_2_. For calcareous macroalgae, we split species into two groups based on prior observations at CO_2_ seep locations and in laboratory manipulation experiments^3^,^23^,^35^. These include group (4) the obligate calcifiers (calcareous reds and greens), and (5) non-obligate calcifiers (calcareous browns, Ochrophyta; *Padina* spp.) which would be grouped based on the characteristics of their CCM).

Macroalgal tissue δ^13^C can be used to determine the presence or absence of a CCM, and δ^13^C can indicate changes in CO_2_:HCO_3_^−^ uptake, when corrected for other factors^39^,^40^. The δ^13^C of CO_2_ and HCO_3_^−^ in seawater is between ~−10 and 0‰^41^. Macroalgal uptake of CO_2_ versus HCO_3_^−^ results in different tissue δ^13^C, with sole CO_2_ uptake via diffusion over the plasmalemma theoretically resulting in values more negative than −30‰, sole HCO_3_^−^ uptake resulting in values less negative than −10‰. Values between −30‰ and −10‰ can result from either diffusive CO_2_ influx, with higher δ^13^C values indicating a large resistance to diffusion with the same CO_2_ concentration difference, or HCO_3_^−^ influx with higher values indicating a smaller fraction of the pumped HCO_3_^−^ leaking out as CO_2._ Therefore, an organism utilising both CO_2_ and HCO_3_^−^ would result in values in δ^13^C values in between −30 and −10. Here we define species mentioned in this study as having evidence for a CCM if δ^13^C > −30‰ and as having evidence of a lack of a CCM if δ^13^C < −30‰. Accumulation of compounds such as lipids could alter tissue δ^13^C values, as transforming photosynthate into lipids could decrease δ^13^C^40^. In past research on phytoplankton, lipid content per cell increased dramatically under elevated CO_2_, and lipids had much lower δ^13^C than the bulk organic matter^42^. The δ^13^C of source seawater DIC could also influence tissue δ^13^C, regardless of CO_2_:HCO_3_^−^ uptake ratios.

Here, we assess whether a mechanistic understanding of macroalgal DIC physiology can be used to predict changes in macroalgal communities along a seawater pCO_2_ gradient at Vulcano, Italy. We hypothesized that (1) at Vulcano the δ^13^C values of all macroalgal species in group 2 (possessing CCMs, with low affinities for CO_2_, or having a downregulated CCM) would increase (i.e. they would be depleted in δ^13^C; evidence for increased reliance on CO_2_) as pH declines; (2) the percentage cover of non-CCM species (group 1) and those CCM species capable of utilising additional CO_2_ (group 2) would increase at all sites as CO_2_ increases; (3) the percentage cover of all green and red calcareous species (group 4) and fleshy macroalgae from group (3) would decrease as CO_2_ increases; and (4) lipid content (a potential influence on δ^13^C) of all species would not differ significantly across locations with differing CO_2_ at Vulcano.

## Results

The abundance of 17 macroalgal species varied significantly along a gradient of CO_2_ (PERMANOVA, *F* = 8.58, *P* < 0.01; Figs 2 and 3). *Udotea petiolata* was not present in the surveys at the pH 8.04 and 7.89 sites, while *Caulerpa prolifera* and *Dilophus fasciola* were not present at the pH 8.04 location. *Dictyota dichotoma* was the species whose abundance increased the most between the pH 8.04 and 7.69 locations (17-fold increase). This was followed by *Caulerpa racemosa* (6 fold increase), *Padina pavonica* (5-fold increase), *Sargassum muticum* (3-fold increase), and *Dictyopteris polypodioides* (2-fold increase). Conversely, crustose coralline algae (CCA) and 8 individual species of macroalgae decreased in abundance between the pH 8.04 and 7.69 locations. CCA had <1% cover at the pH 7.69 and 7.89 locations, while *Halopteris scoparia, Cystoseira crinita, C. barbarta* and *C. brachycarpa* had <1% cover at the pH 7.69 location. Four other species decreased in abundance between the pH 8.04 and 7.69 locations. In order from the largest to the smallest decrease in percent cover these were *Cystoseira foeniculacea, Codium bursa, Cystoseira compressa*, and *Acetabularia acetabulum*. Most species made minor contributions to the percent cover overall, except *Cystoseira foeniculacea* and *Dictyota dichotoma* which showed opposing shifts in abundance between locations (Fig. 2). *C. foeniculacea* had the highest percent cover at the pH 8.04 (44.78% cover) and pH 7.89 (24.98%) locations, but was the second most abundant species at the pH 7.69 location (8.46%). *D. dichotoma* had the highest abundance at the pH 7.69 location (40.14% cover), the 2^nd^ highest at pH 7.89 (14.04%) and the 9^th^ highest at pH 8.04 (2.33%).

Species possessing CCMs dominated percent cover at Vulcano at all locations. However, there were noticeable differences between the percent cover of physiological groups between locations (PERMANOVA: *F* = 12.29, *p* < 0.01, Figs 3 and 4). Non-CCM (*Udotea petiolata*) cover was highest at the pH 7.69 location, calcareous brown (*Padina pavonica*) highest at pH 7.89, and the cover of calcareous reds (corallines and *Peyssoniellia* spp.) were lowest at pH 7.89 and 7.69 locations (Fig. 3a). We grouped the species with δ^13^C values that significantly decreased as pH declines as low affinity CCM species (group 2 – see the introduction), and those with δ^13^C values that did not vary significantly by pH location as high affinity species (group 3). We then test whether there are differences in the composition of species that theoretically should benefit from elevated CO_2_ (groups 1 and 2) or not (groups 3 and 4). Results indicate that there are significant differences in their distribution at the three locations at Vulcano (PERMANOVA: *F* = 17.22, *p* < 0.01, Fig. 3a). The abundance of species predicted to increase between pH 8.04 and pH 7.69 did so, and those predicted to decline in abundance between pH 8.04 and 7.69 also did (Fig. 3a).

Shifts in species percent cover were related to changes in tissue δ^13^C, whereby species whose abundance increased by the largest amount as pH declined also tended to be the same species whose tissue δ^13^C was significantly depleted. We observed significant declines in δ^13^C at elevated CO_2_ locations in 5 species (Table 1). Only one species (*Udotea petiolata*) had δ^13^C ~−30‰ (Table 1), indicating the possible lack a CCM. Five of six species that were found in greater cover at elevated CO_2_ locations had δ^13^C values that were more depleted at elevated CO_2_ locations (*Caulerpa prolifera, Dilophus fasciola, Dictyota dichotoma, Caulerpa racemosa*, and *Padina pavonica*). All five species that declined in abundance at elevated CO_2_ locations had no significant differences in δ^13^C values. For *D. polypoides*, there was insufficient material collected at the pH 8.04 location to analyse δ^13^C values. Our seawater DIC δ^13^C values were relatively similar across all three locations, so we use the raw δ^13^C values of the macroalgae, rather than correcting for source seawater DIC δ^13^C.

Lipid concentrations did not vary among species examined, and did not vary by mean CO_2_ at the three locations at Vulcano (SI 1). The presence of a CCM was confirmed for *Sargassum muticum, Halopteris scoparia, Padina pavonica* and *Cystoseira foeniculacea* by pH drift experiments that showed pH compensation points above 9 at all locations for all species. Only for *Cystoseira foeniculacea* was there a significant difference between final pH compensation points between sites (SI 2).

To test the generality of our findings, we examined changes in the abundance of physiological groups of macroalgae at CO_2_ seeps off Ischia, Italy, and Methana, Greece. We combined published species abundances^30^,^43^ and δ^13^C values (SI 3) to assess changes in the five physiological groups. In Ischia, non-CCM abundance also increased at the elevated CO_2_ location, and calcareous red species cover declined. At Methana there were significant shifts in the physiological groups (PERMANOVA, *F* = 1.63, *p* = 0.04), with calcareous red macroalgae declining as CO_2_ increased (Fig. 3c). As for Vulcano, we also grouped species abundances based on whether they were predicted to benefit from elevated CO_2_ or not. At Methana, we see significant differences in the abundance of species predicted to benefit from elevated CO_2_ or not between the three different pH locations (PERMANOVA, *F* = 5.06, *p* = 0.02). The abundance of species not predicted to benefit from elevated CO_2_ were lowest at the pH 7.67 location, and the abundance of species predicted to benefit were highest at the pH 7.67 location. However, these changes were not as large as at Vulcano (Fig. 3), and there was some seasonality to these effects, with groups predicted to benefit from elevated CO_2_ being associated more with pH 7.67 locations in spring than in autumn (Fig. 4c). Species from combined groups 1 and 2 tended to increase in abundance at pH 7.80 at Ischia, whereas species from the combined groups 3 and 4 decreased in abundance relative to the pH 8.10 location. However, the data from Ischia could not be analysed statistically, as we did not have the raw data, only the mean of each location.

## Discussion

**Figure 1:**
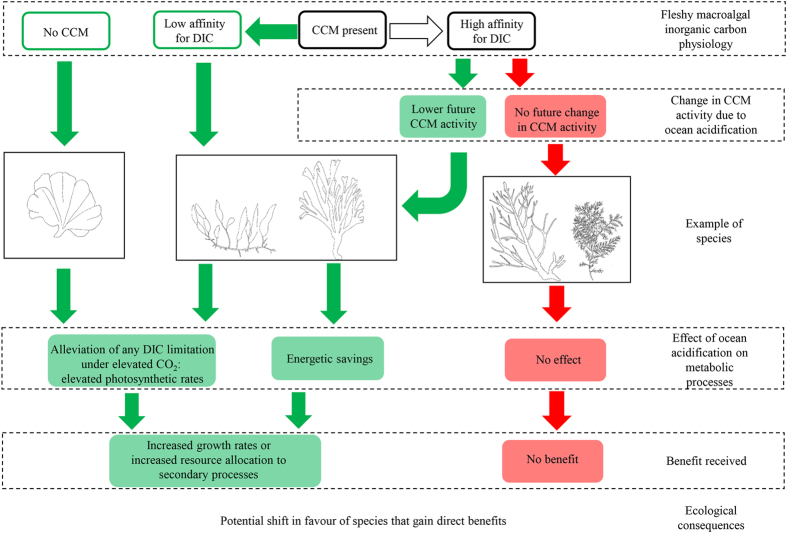
Responses of fleshy macroalgae to ocean acidification in relation to their CO_2_ concentrating mechanisms (CCMs), showing the different physiological groups defined here, mechanisms defining how increased CO_2_ could directly affect their physiology, and higher order flow-on effects. Pictured are species which represent those with no CCM (*Udotea petiolata*), low affinity CCM/lower future CCM activity (*Caulerpa prolifera* and *Dictyota dichotoma*) and high affinity CCM with no change in future CCM activity under ocean acidification (*Halopteris scoparia* and *Cystoseira foeniculacea*).

Predicting changes in the structure and dynamics of future macroalgae assemblages due to the direct effects of ocean acidification requires an understanding of the physiological mechanisms of DIC acquisition. The majority of macroalgal species at Mediterranean seep sites had CCMs, and the direction of the change in their abundance at elevated pCO_2_ at Vulcano was correlated with whether or not their δ^13^C values decreased: species with decreased δ^13^C were more likely to increase in abundance and vice versa. This decrease of δ^13^C with increasing CO_2_ most likely resulted from an increase in the reliance on CO_2_ during photosynthesis; we discuss other possibilities below. Species utilising additional CO_2_ would either receive benefits through the alleviation of DIC limitation, or through the down-regulation of energetically expensive CCMs (Fig. 1). This supports predictions that macroalgal species capable of utilising additional CO_2_ (i.e. non-CCM species and CCM species with low affinities for DIC) will benefit from ocean acidification, while species that cannot (high affinity CCMs with no down-regulation)^18^,^32^, and obligate calcifying species, will decrease in abundance. Although only one non-CCM species was present in our surveys at Vulcano, surveys at other Mediterranean CO_2_ seep sites support our suggestion that fleshy macroalgae with a predictable suite of physiological traits are able to benefit from elevated CO_2_ over species that have physiologies poorly suited to higher CO_2_ concentrations.

The dominance of fleshy macroalgae and absence of calcifying macroalgae at vent sites with high CO_2_ is well known^28^,^30^,^43^,^44^, and this study reveals for the first time that these patterns in macroalgal assemblages, which are consistent across several vent sites, are correlated with the DIC physiology of specific species. Macroalgae that thrived at locations with high CO_2_ at multiple sites were *Dictyota* spp. (4/4 sites)^28^,^30^,^43^,^45^, *Caulerpa* spp. (3/3 sites)^28^,^45^, *Udotea petiolata* (2/3 sites)^43^, and *Sargassum* spp. (4/4 sites)^28^,^30^,^43^. This includes two CCM genera containing species whose δ^13^C values indicate higher reliance on CO_2_ at Vulcano (*Dictyota* and *Caulerpa*), one non-CCM species (*U. petiolata*), and one CCM species whose δ^13^C values did not change at Vulcano (*Sargassum* spp.). Macroalgae that had reduced percent cover at high CO_2_ locations included coralline algae^28^,^30^,^43^,^45^ (4/4 sites), calcifying green algae^28^ (2/2 sites), some *Cystoseira* spp. (3/4 sites)^30^,^43^,^45^ and *Halopteris scoparia* (2/2 sites)^43^. This includes two calcifying groups (coralline algae and calcifying green algae) and two CCM genera with species whose δ^13^C values did not indicate higher reliance on CO_2_ at Vulcano. These trends in changing species abundances further support the hypothesis that species responses to ocean acidification will be influenced by their DIC physiology^18^. The direct effect of changing CO_2_ concentrations on macroalgal physiology is important, but it is likely that changes in species abundances due to ocean acidification will also be modified by shifting species interactions^8^,^9^ and by changes in other environmental factors or their variability^46^,^47^,^48^, such as light^18^,^49^.

Natural CO_2_ seeps are useful in determining the structure of marine assemblages at elevated CO_2_, but they are not perfect analogues for the effects of ocean acidification. As mean CO_2_ concentrations increase, variability about the mean also increases near the seeps which may exacerbate any negative effects. Coralline algae, for example, are particularly vulnerable to rapid declines in carbonate saturation/increases in proton concentrations^20^,^21^,^50^. Therefore, the effects of mean increases in CO_2_ concentrations due to ocean acidification on macroalgal assemblages could differ from the effects of volcanic acidification examined here. Other physicochemical gradients, such as water motion, may affect organisms near volcanic seeps. Although our locations at Vulcano were wave-sheltered, there was a small increase in exposure moving away from the seeps. This gradient of water motion would be expected to act on macroalgal δ^13^C in the reverse direction to CO_2_ because thinner diffusion boundary layers result in greater uptake of CO_2_ at more wave exposed locations^39^. Therefore, our results are conservative, as the decrease in macroalgal tissue δ^13^C would be greater if this gradient in wave exposure did not exist.

We consider that the decreased δ^13^C values of specific macroalgae from elevated CO_2_ locations is most likely caused by changes in the ratio of CO_2_:HCO_3_^−^ uptake. However, there are other mechanisms unrelated to increased reliance on CO_2_ that would result in decreased tissue δ^13^C. The most likely scenarios are: (1) Increases in the phosphoenolpyruvate carboxykinase (PEPCK)-based C4 pathways could result in decreased δ^13^C^51^,^52^, and for *Udotea petiolata* this could explain the <−30‰ values; a phosphoenolpyruvate carboxylase (PEPC)-based C4 pathway would result in less negative δ^13^C values^51^,^53^,^54^. (2) Lower photosynthetic rates (resulting in lower uptake of ^13^C enriched HCO_3_^−^) by macroalgae would also result in decreased δ^13^C at elevated CO_2_. As these species tended to increase in abundance at Vulcano, their photosynthetic rates would not be expected to decline. Indeed, past research has found elevated photosystem II relative electron transport rates (used an indicator of photosynthesis *in situ* in that study) of *Padina pavonica* at elevated CO_2_ locations in Vulcano^35^. (3) Leakage of CO_2_ from the CCM decreasing as concentrations of CO_2_ increases externally, with CCM pumping of DIC into the cell remaining equal^55^. Apart from decreased leakage, none of these scenarios would explain why the species of macroalgae with depleted δ^13^C also tended to have higher cover at elevated CO_2_ locations throughout the Mediterranean, as most of these alternate explanations would not provide benefits. Similar decreases in tissue δ^13^C as CO_2_ concentrations increase has also occurred for other macroalgal^20^,^56^,^57^, seagrass^58^ and phytoplankton species^59^. However, these changes did not always correspond to changes in rates of photosynthesis, DIC acquisition, or growth. For freshwater macroalgae, decreased δ^13^C and a higher abundance of non-CCM species were also found in locations with elevated CO_2_^60^, further supporting our conclusions. While further research is still required to examine *in situ* photosynthetic rates of macroalgae at seep sites and to determine the relationship between δ^13^C and CCM activity, we conclude based on the evidence available that increased uptake of CO_2_ during DIC acquisition as mean CO_2_ concentrations increase is the most likely and logical cause of decreased tissue δ^13^C in our study.

The effects of ocean acidification on macroalgal assemblages will likely depend on the natural assemblage composition in a particular region. For example in the Mediterranean and in coral reef ecosystems, the abundance of non-CCM species is low^61^,^62^ compared to temperate subtidal ecosystems^18^,^63^, meaning that greater changes in species compositions due to ocean acidification could occur in ecosystems possessing more non-CCM species, compared to those trends observed here. CCM species are relatively abundant in most shallow-water marine ecosystems^18^,^63^, particularly in warmer climates (such as in this study and on the Great Barrier Reef ref. 61), and the majority of macroalgal species in rocky and coral reefs possess CCMs^18^,^61^,^62^,^63^. Therefore, the direct effects of ocean acidification on macroalgae could manifest via increases in the abundance of low-affinity CCM species in most ecosystems dominated by macroalgae, particularly warmer locations.

Here we demonstrate a relationship between the DIC physiology of macroalgae and their changes in abundance at natural CO_2_ seeps in the Mediterranean. Predicting changes in macroalgal communities due to ocean acidification is complex. This is in part because we do not understand the relative roles of direct and indirect effects of ocean acidification in shaping macroalgal assemblages. In ecosystems where strong top-down control exists, responses of benthic communities could be more related to changes in higher trophic levels^8^,^9^, but when top-down control is relatively weak, responses could be dictated primarily by macroalgal DIC physiology. Additionally, the direct effects of ocean acidification could be modified by the presence of competitors or facilitators^17^,^64^,^65^. The challenge now is to determine whether the physiological groups defined here respond similarly elsewhere. It is clear is that most calcareous macroalgae will be negatively impacted by elevated mean CO_2_, but that species which are not obligate calcifiers (such as *Padina pavonica*) are tolerant^3^,^14^,^20^,^35^,^48^,^66^. Fleshy macroalgae respond positively if they are reliant only on CO_2_, or if their reliance increases as CO_2_ concentrations increase. The capacity to utilise additional seawater CO_2_ means that non-CCM species, or species with CCMs with lower affinities for DIC, will likely benefit from ocean acidification.

Currently, there is a limited understanding of how macroalgal physiology could be used to predict general shifts in abundance or fitness under ocean acidification. This is due to the complex nature of macroalgal inorganic carbon physiology and calcification, and in part, the difficulties in thoroughly assessing the DIC physiology of numerous taxa from different geographic regions. For example, the traditional approach of examining changing photosynthetic rates over constant additions of DIC is problematic, because there is the possibility that future seawater could favour the retention of a CCM in macroalgae that are not DIC-saturated today^67^. However, this would only occur until the external seawater CO_2_ concentrations exceeded that produced internally by the CCM, so that replacement of the CCM with diffusive CO_2_ entry to Rubisco could occur and it is unknown whether the additional CO_2_ or HCO_3_^−^ relieves this DIC limitation. Greater progress has been made determining mechanistically why the calcification of some macroalgal species may be impacted by declining pH and increasing CO_2_ concentrations^15^,^68^,^69^,^70^,^71^, although more work is required^72^. Our study provides an essential stepping stone to improving knowledge regarding how fleshy macroalgae might respond in a future elevated CO_2_ ocean, as the first general test of how macroalgal DIC physiology is linked to responses to natural acidification *in situ*. Our framework explains why elevated CO_2_ does not ubiquitously alter habitat types at CO_2_ seep sites between canopy-forming and turf species^29^: species compositions will change depending on resident species’ physiology, which is only sometimes related to their morphology or functional form group. From knowledge of the DIC physiology of macroalgae within a community, an improved framework can be created by which we can project the responses of coastal marine assemblages to ocean acidification.

## Materials and Methods

In late May to early June 2014 we surveyed pH_T_ (pH on the total scale, hereafter “pH”), total alkalinity (hereafter “A_T_”), DIC, and δ^13^C of DIC at three locations with a mean pH of 8.04, 7.89 and 7.69 at 2 m depth (“pH 7.69” 38°25.176′N, 14°57.658′E; “pH 7.89” 38°25.193′N, 14°57.763′E; “pH8.04” 38°25.248′N, 14°57.853′E; see SI 6) where pH, A_T_ and some associated biogeochemical parameters have been monitored for six years^35^,^73^,^74^. For long term pH data and other environmental variables (e.g. H_2_S, total alkalinity and heavy metals) at this site see Boatta *et al*.^74^. Surveys of macroalgal abundance were conducted on the 26 May 2014, and involved three 25 m transects at each of the 3 locations. Each transect involved ten 50 by 50 cm photoquadrats, from which the percent cover of individual species were determined. *Cystoseira* spp. were lumped into morphological categories and later identified to species were possible. Macroalgae were grouped based on their use of DIC into non-CCM, CCM, and calcareous species based on δ^13^C values (after ref. 18). Calcareous species were further split into reds, greens and browns because these taxonomic groups have different calcification mechanisms^75^, which may influence their response to ocean acidification^35^,^66^.

Samples of each species were collected from the three locations for δ^13^C analysis. Due to the scarcity of some species at specific locations, not all species used here were collected at all three locations. A minimum of three replicates were collected of each species at each location where possible, with each replicate being a different individual and a total of 19 species being analysed. All samples were air dried at 60 °C for 24 hours. Then they were ground into a fine powder using a porcelain mortar and pestle and weighed in to pressed tin capsules (0.2 mg; Sercon, UK). Isotope values were determined using a Fisons NA1500 elemental analyzer coupled to a Thermo Scientific Delta V Plus via a Conflo IV. Combustion and reduction were achieved at 1020 °C and 650 °C respectively. Percent C and N composition was calculated by comparison of mass spectrometer peak areas to those of standards with known concentrations. Isotopic values for carbon are reported as δ-values (‰) relative to Vienna Pee Dee Belemnite (VPDB) and were corrected via a 4-point calibration using certified standards. Reproducibility was also monitored by the use of long term internal reference materials. Both precision and accuracy were ±0.1‰ (1 SD). All samples were run in duplicate and averaged when values differed.

The four most abundant and easily identifiable species – all of which were present at all sites – were collected on May 27^th^ from each station for simultaneous pH drift assays. pH drift assays determine whether a species is capable of utilizing HCO_3_^−^ by assessing its capacity to elevate pH above 9 – the point at which CO_2_ concentrations are functionally zero. For more details see refs 49,76,77. Specimens were placed into 50 ml transparent plastic containers that were maintained in a water bath for 24 hours. Water in the water bath was manually replaced every 30 minutes during daylight hours to maintain temperatures within 1.5 °C of the ambient seawater temperature (24 °C). Seawater pH was measured using a combined pH and RDO meter (Orion Star A216 pH/RDO/DO) and pH electrode (Orion 8107BNUMD – Ross Ultra pH/AIC triode), calibrated with pH 7.0 and 9.0 NBS buffers, cross referenced with Tris and Amp seawater buffers to convert to the total scale^78^. Oxygen was measured using an Orion RDO probe (ORI087100MDW) attached to the same meter, and was calibrated using 0% and 100% air saturation standards made by bubbling seawater with N_2_ or air respectively for 10 minutes.

Seawater pH was measured at all three locations during the study duration on three days, and involved triplicate discrete measurements using the electrode noted above. Samples for A_T_ and DIC were taken on June 1^st^ from all three locations. Seawater was stored in 250 ml glass containers and poisoned immediately with HgCl_2_. A_T_ was measured using an open-cell titration using a Metrohm 809 Titrando and Metrohm 800 Dosino. A_T_ measurements were made at 25 °C using a Circu temperature bath, while DIC was measured using a LiCOR DIC analyser. Both were measured against a CRM supplied by Andrew Dickson (see ref. 78). Measured accuracy and precision of the CRM was within ±5 μmol kg^−1^ for both DIC and A_T_, within the standard error of the differences between measurements from any one location. SI 6 lists the mean seawater carbonate chemistry at the three locations. δ^13^C of seawater DIC was determined using a Gasbench II coupled to a Delta V Plus via a Conflo IV (Thermo Scientific; after ref. 79). Samples (0.5 mL) were injected into helium flushed 12 mL septum capped exetainers (Labco, High Wycombe, UK) containing phosphoric acid (85%). Samples were equilibrated overnight after which time liberated CO_2_ is passed to the IRMS in a stream of helium passing through a nafion water separator and a Poroplot Q GC column (25 m × 0.32 mm ID, 45 °C, 2.5 mL/min). Isotopic values for carbon are reported as δ-values (‰) relative to VPDB and were corrected via a 2-point calibration using certified standards. Reproducibility of replicates was 0.05‰. Four replicates samples were collected from each site, but half of these were broken by Australian Customs for the pH 7.89 and 7.69 sites. All replicate samples were then measured in duplicate.

Lipid content of macroalgae was measured by extracting samples quantitatively overnight using a single-phase dichloromethane/methanol/water mixture (after ref. 80). Following phase separation, lipids were recovered in the lower dichloromethane layer and concentrated by rotary evaporation to provide the total lipid extract. This was transferred to pre-weighed glass vials using solvent which was subsequently removed under a stream of nitrogen allowing lipid weight to be determined.

**Figure 2:**
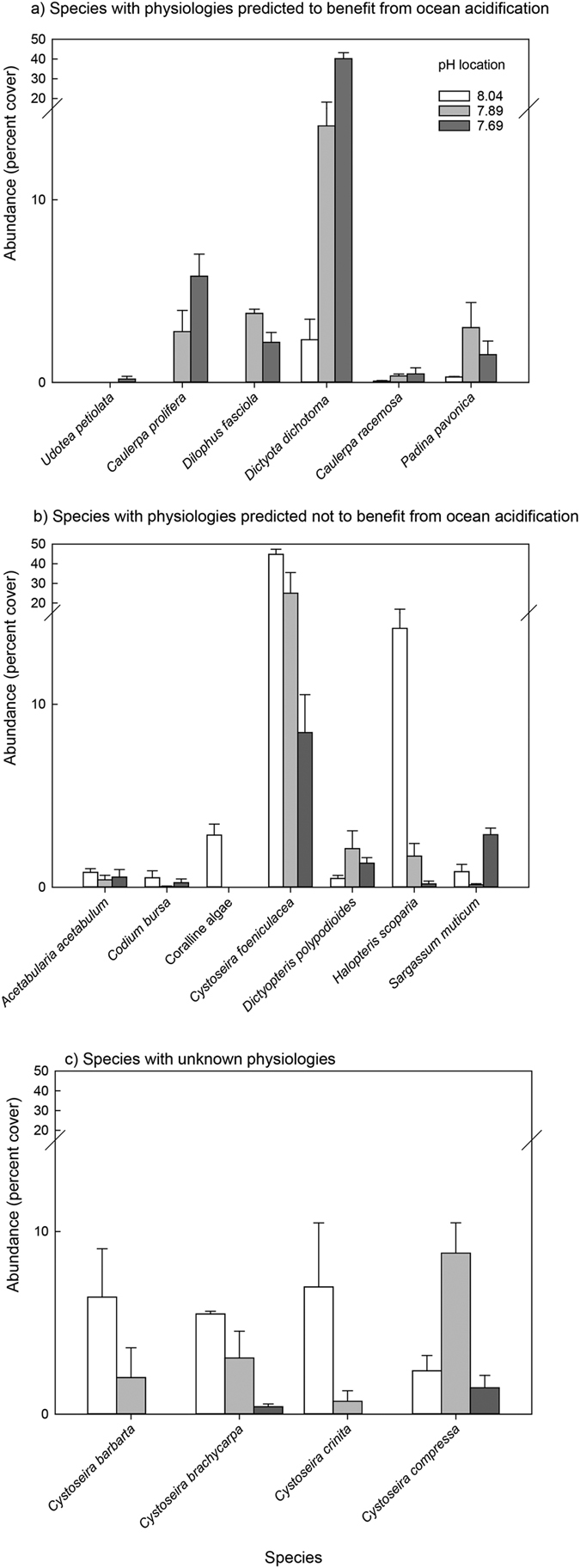
Percentage cover of macroalgal species from locations with three mean seawater pH values at Vulcano, Italy at three locations differing in mean seawater pH. (**a**) Species with physiologies predicted to benefit from elevated CO_2_ concentrations. (**b**) Species with physiologies predicted to not benefit from elevated CO_2_ concentrations. Each bar represents mean ±1 s.e. *n* = 3 transects per site and 10 quadrats per transect.

**Figure 3:**
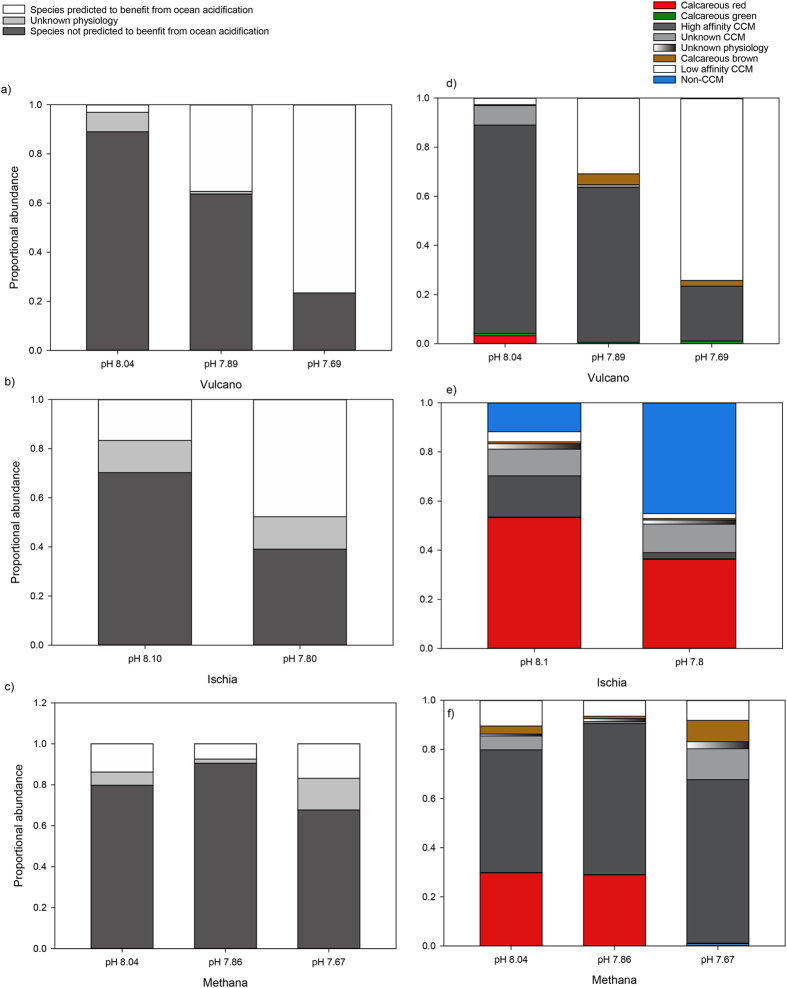
Proportional cover of species predicted to benefit from elevated CO_2_, those with unknown physiologies, and those predicted not to benefit from elevated CO_2_ at three different CO_2_ seep sites: (**a**) Vulcano, (**b**) Ischia, and (**c**) Methana. Proportional cover of the physiological groups: non CCM (CO_2_ concentrating mechanism), unknown (possible non-CCM), high affinity CCM, low affinity CCM, unknown CCM, calcifying reds, greens and browns at CO_2_ seep locations with different mean seawater pH. (**d**) Vulcano. (**e**) Ischia. (**f**) Methana. See methods for more details.

**Figure 4:**
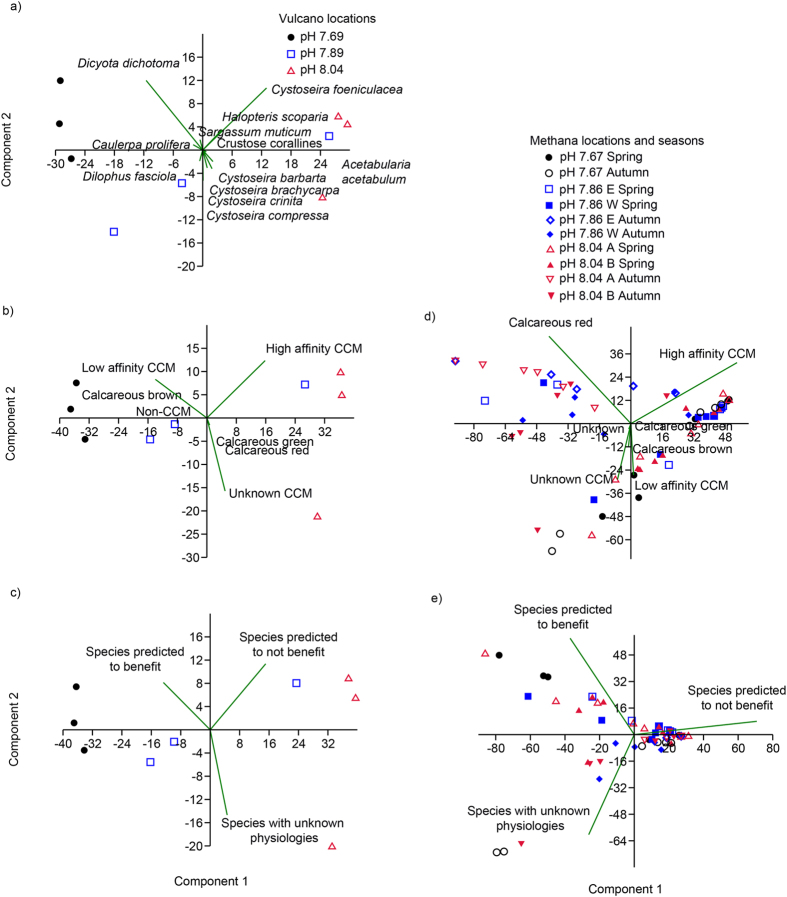
PCA plot displaying the association of different species at Vulcano (**a**), physiological groups at Vulcano (**b**) and Methana (**d**), and winners and losers from Vulcano (**c**) and Methana (**e**) across the different transects at both sites at locations with different mean seawater pH.

Most CO_2_ seeps have elevated H_2_S or toxic metals so detailed geochemical monitoring is needed to rule out these potentially confounding factors (e.g. at Ischia) or minimize their likely effects (e.g. at Vulcano)^74^. The effects of elevated CO_2_ at individual seep sites could be altered by other local factors such as wave exposure, nutrient concentrations or turbidity. Additionally, nature is inherently variable, and observations of changing patterns in species’ abundances at one location may only represent snapshots of variability in species’ abundances in time in space^81^. For these reasons we compared the patterns we observed along the Vulcano gradient with published studies on macroalgal abundances at CO_2_ seep sites off Ischia (Italy) and Methana (Greece). To calculate the percent cover of the five different physiological groups of macroalgae, percent cover data from Porzio *et al*.^43^ (Ischia) and Baggini *et al*.^30^ (Methana) were used and macroalgae were grouped based on their use of DIC. For fleshy species, δ^13^C from this study or from the literature were used to determine CCM presence or absence. SI 5 lists the details of this analysis beyond those presented in Fig. 2.

ANOVA was used to examine differences in δ^13^C and final O_2_ concentrations and pH in pH drift experiments between locations within species. All data were checked for normality and heterogeneity first, and passed all checks. When statistical differences were found in ANOVAs, Tukey’s post-hoc tests further determined differences between particular locations along the CO_2_ gradient. These analyses were conducted in R v. 3.20. Differences in percent cover of species (Vulcano only), the physiological groups, and the species we predict to benefit or not from elevated CO_2_ between the locations with differing CO_2_ concentrations where analysed using separate PERMANOVAs in PAST for each site (Vulcano and Methana, i.e. our study site and that published previously by Baggini *et al*.^30^). Methana was the only additional site analysed using PERMANOVA and PCA because it was the only site where published raw data was available that contained species abundances, and not morphologically grouped species.

## Additional Information

**How to cite this article**: Cornwall, C. E. *et al*. Inorganic carbon physiology underpins macroalgal responses to elevated CO_2_. *Sci. Rep.* **7**, 46297; doi: 10.1038/srep46297 (2017).

**Publisher's note:** Springer Nature remains neutral with regard to jurisdictional claims in published maps and institutional affiliations.

## Supplementary Material

Supplementary Information

## Figures and Tables

**Table 1:** Mean ± 1 s.e. of δ^13^C of macroalgal tissue at the three locations at Vulcano, Italy, with varying mean seawater pH values.

Species	pH 7.69	pH 7.89	pH 8.04	*F* value (d.f)	*P* value	Physiological group
	δ^13^C	δ^13^C	δ^13^C			
*Acetabularia acetabulum*	−10.4 ± 0.3	−10.8 ± 0.1	−11.1 ± 0.5	0.67_(2,6)_	0.55	Calcifying green
***Caulerpa prolifera***	−16.1 ± 1.3	−15.4 ± 0.8	−13.2* ± 1.1	5.19_(2,6)_	**0**.**04**	Low affinity CCM
***Caulerpa racemosa***	−17.0 ± 0.2	−15.2 ± 0.2		19.06_(1,4)_	**0**.**01**	Low affinity CCM
*Codium bursa*	−20.1 ± 0.2	−21.0 ± 0.1	−22.5 ± 2.1	1.02_(2,6)_	0.42	High affinity CCM
*Cystoseira amentacea*		−24.6				Unknown CCM
*Cystoseira brachycarpa*		−18.3 ± 0.3				Unknown CCM
*Cystoseira compressa*	−20.5 ± 0.2		−18.8 ± 1.3	3.53_(1,4)_	0.11	High affinity CCM
*Cystoseira compressa* 2013	−12.6 ± 0.28	−14.2 ± 0.5	−13.8 ± 0.5	1.02_(2,6)_	0.42	High affinity CCM
*Cystoseira foeniculacea*	−18.8 ± 1.7	−19.5 ± 0.9	−20.0 ± 1.2	0.29_(2,7)_	0.76	High affinity CCM
*Dictyopteris polypodioides*	−19.8 ± 0.3	−19.5 ± 0.7		0.09_(1,4)_	0.78	High affinity CCM
***Dictyota dichotoma***	−24.8 ± 0.3		−22.0 ± 0.1	24.66_(1,4)_	<**0**.**01**	Low affinity CCM
***Dictyota dichotoma*** **2013**		−18.9 ± 0.6	−15.7 ± 1.1	7.05_(1,5)_	**0**.**04**	Low affinity CCM
***Dilophus fasciola***	−22.1 ± 0.1	−18.4 ± 1.3		11.82_(1,4)_	**0**.**03**	Low affinity CCM
*Dilophus fasciola* 2013	−15.5 ± 0.3					Low affinity CCM
*Halopteris scoparia*	−22.2 ± 0.8	−21.0 ± 0.5	−21.9 ± 0.3	3.22_(2,6)_	0.09	High affinity CCM
*Jania rubens*	−20.2 ± 1.8		−22.1 ± 0.5	2.81_(1,4_)	0.17	Calcareous red
***Padina pavonica***	−14.9_a_ ± 0.4	−16.3_b_ ± 0.3	−13.5_c_ ± 1.0	6.50_(2,6)_	**0**.**03**	Calcareous brown
***Padina pavonica*** **2013**	−14.0_a_ ± 0.2	−12.7_b_ ± 0.1	−12.0_c_ ± 0.2	31.44_(2,12)_	<**0**.**01**	Calcareous brown
*Peyssonnelia squamaria*	−20.3		−23.0			Calcareous red
*Sargassum muticum*	−19.6 ± 0.2	−19.3 ± 1.1	−19.6 ± 0.1	0.08_(2,7)_	0.92	High affinity CCM
*Sargassum muticum* 2013	−19.8 ± 0.3	−20.6 ± 0.7	−19.2 ± 0.3	2.27_(2,12)_	0.14	High affinity CCM
*Tricheocarpa fragilis*	−9.0		−9.6 ± 0.6			Calcareous red
*Udotea petiolata*	−30.5 ± 0.2	−30.3 ± 0.7		0.03_(1,4)_	0.86	Non-CCM

Analysis of variance (ANOVA) results of the effect of pH location on δ^13^C. Species names in bold indicate those with *P* values lower than 0.05, values with asterisks next to them indicate these values were significantly different to all other sites, while lowercase subscript letters indicate significant difference between individual sites during Tukey’s post-hoc analysis.
