# Supplementary material for: Inorganic carbon physiology underpins macroalgal responses to elevated CO2
Source: Sci Rep. 2017 Apr 18;7:46297. doi: 10.1038/srep46297 (PMC5394685; doi:10.1038/srep46297)
Supplement: Supplementary Information [file srep46297-s1.pdf]

Supplementary information for:

Inorganic carbon physiology underpins macroalgal responses to elevated CO_2

Christopher E. Cornwall[1,2,*], Andrew T. Revill[3], Jason M. Hall-Spencer[4,5], Marco Milazzo[6], John A. Raven[7,8], Catriona L. Hurd[1]

[1]Institute for Marine and Antarctic Studies, University of Tasmania, Hobart, Tasmania 7001, Australia

[2]School of Earth and Environment and ARC Centre of Excellence for Coral Reef Studies, University of Western Australia, Crawley, Western Australia 6009, Australia

[3]CSIRO Oceans and Atmosphere, Hobart, Tasmania 7000, Australia

[4]Marine Biology and Ecology Research Centre, Plymouth University, Plymouth, UK

[5]Shimoda Marine Research Centre, University of Tsukuba, Japan

[6] DiSTeM, CoNISMa, University of Palermo, Palermo, Italy

[7] Division of Plant Science, University of Dundee at the James Hutton Institute, Invergowie, Dundee, DD2 5DA, UK

[8]School of Plant Biology, University of Western Australia, Crawley, Western Australia 6009, Australia

[*]Corresponding author. Email: Christopher.cornwall@uwa.edu.au; phone: + 61 8 6488 3644

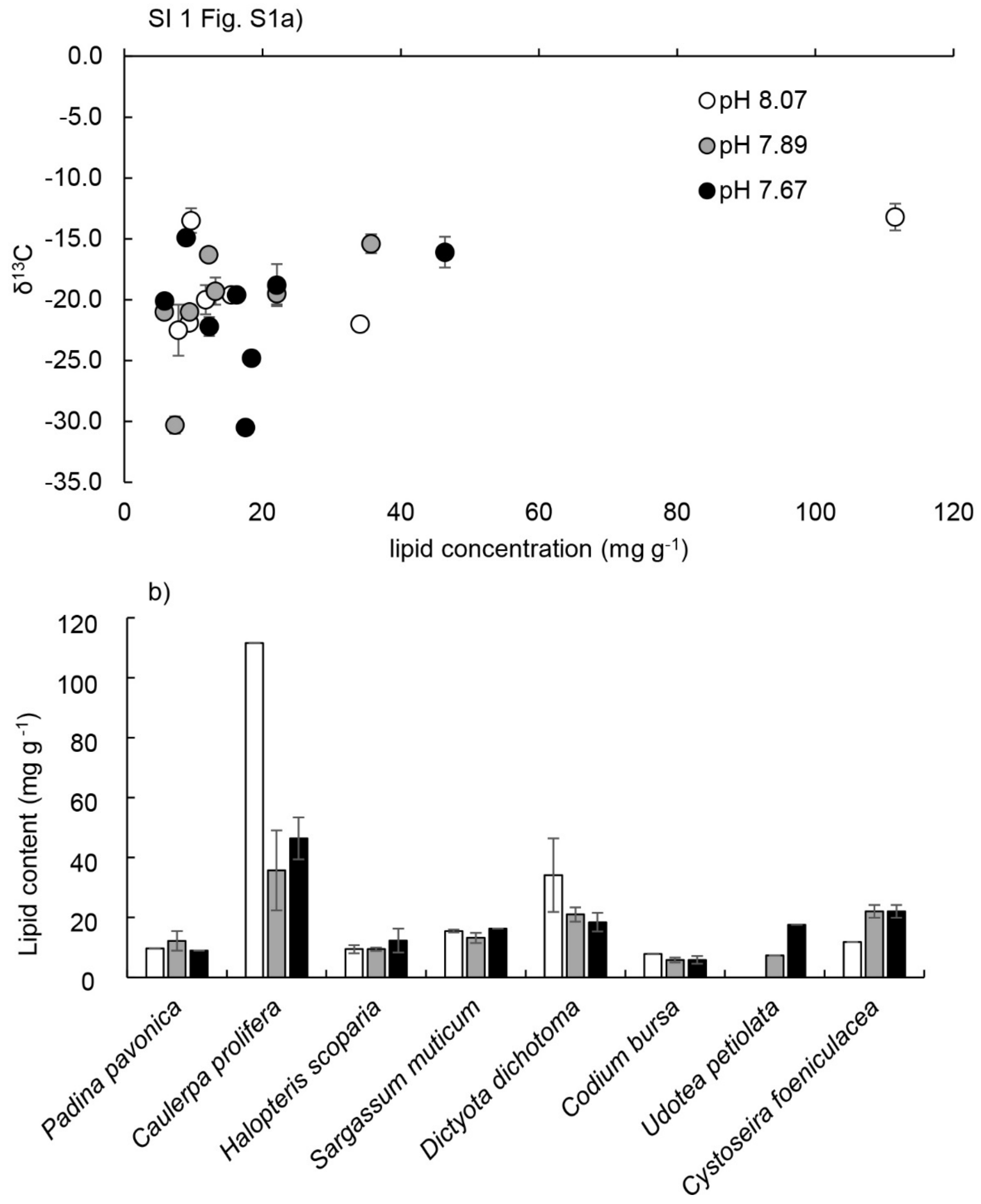

SI 1, SI Fig. 1: a) Concentrations of lipids plotted against $\delta^{13}C$ and b) species' mean lipid content of macroalgae collected at locations with differing seawater pH at a CO_2 seep from Vulcano, Italy, showing no relationship between lipid concentration and $\delta^{13}C$. $n = 3 – 4$. Mean ± one standard error.

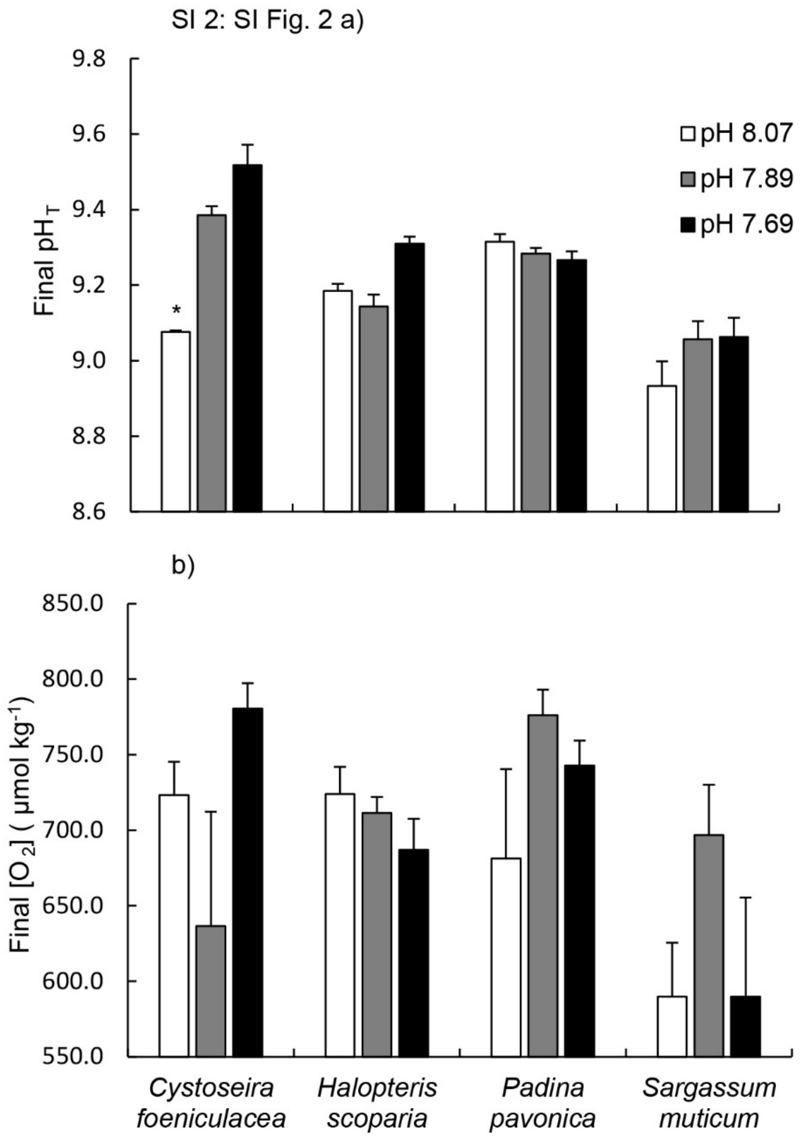

SI 2, SI Fig. 2: a) pH and b) O_2 compensation points of the four most abundant macroalgal species collected at locations with differing seawater pH at a CO_2 seep at Vulcano, Italy, demonstrating only for *Cystoseira foeniculacea* was there a significant difference between the pH compensation points between locations. $n = 3 – 4$. Mean ± one standard error.

SI 3: List of species from Porzio et al. [1] and Baggini et al. [2], and the $\delta^{13}C$ value of that species from literature and its source. When $\delta^{13}C$ for a particular species could not be found, but it could be found for another species in the genus, the other species $\delta^{13}C$ was used: these are marked with *.

Species	$\delta^{13}C$ value from literature	Source of $\delta^{13}C$	Physiological group
Acrothamnion preissii	Unknown		Unknown
Aglaothamnion bipinnatum	-28.96*	[3]	Non-CCM
Aglaothamnion diaphanum	-28.96*	[3]	Non-CCM
Amphiroa criptarthrodia	-13.70 to -13.78*	[4,5]	Calcareous red
Amphiroa rigida	-13.70 to -13.78*	[4,5]	Calcareous red
Amphiroa sp.	-13.70 to -13.78*	[4,5]	Calcareous red
Anotrichium tenue	-30.00 to 31.37*	[6]	Non-CCM
Antithamnion cruciatum	-27.77 to -27.81*	[7]	Unknown
Antithamnion sp.	-27.77 to -27.81*	[7]	Unknown
Bryopsis plumosa	-19.0 to -19.35	[4,8]	CCM
Caulerpa racemosa	-15.20 to -17.50	This study	CCM
Centroceras clavulatum	Unknown		Unknown
Ceramium circinatum	-12.39 to -21.68*	[9]	CCM
Ceramium codii	-12.39 to -21.68*	[9]	CCM
Ceramium diaphanum	-12.39 to -21.68*	[9]	CCM
Ceramium flaccidum	-12.39 to -21.68*	[9]	CCM
Chaetomorpha linum	-14.73 to -21.80	[3,10]	CCM
Champia parvula	-20.33 to -22.82	[7]	CCM
Chondracanthus acicularis	-11.10	[11]	CCM
Chondrophycus papillosus	Unknown		Unknown
Choreonema thuretii	Unknown		Calcareous red
Cladophora dalmatica	-10.83 to -35.57*	[3,12]	Unknown
Cladophora flexuosa	-10.83 to -35.57*	[3,12]	Unknown
Cladophora laetevirens	-15.64	[11]	CCM
Cladophora pellucida	-10.83 to -35.57*	[3,12]	Unknown
Cladophora prolifera	-17.50	[13]	CCM
Cladophora rupestris	-13.33 to -21.30	[8,14]	CCM
Cladophora sericea	-16.66	[8]	CCM
Cladophora socialis	-10.83 to -35.57*	[3,12]	Unknown
Cladophora sp.	-10.83 to -35.57*	[3,12]	Unknown
Cladostephus spongiosus	-16.96 to -21.44	[3]	CCM
Contarinia squamariae	Unknown		Unknown
Corallina elongata	-19.70 to -23.60	[14,15]	Calcareous red
Corallina officinalis	-7.45 to -15.81	[3,11]	Calcareous red
Corallina sp.	-5.69 to -23.60*	[3,15]	Calcareous red
Corallinaceae	-5.69 to -23.60*	[3,15]	Calcareous red
Crouania attenuata	Unknown		Unknown
Cystoseira amentacea	-24.6	This study	CCM
Cystoseira corniculata	-12.60 to -24.60*	This study	CCM
Dasya baillouviana	-15.85 to -34.29*	[4,16]	Unknown

Dasya hutchinsiae	-15.85 to -34.29*	[4,16]	Unknown
Dasya rigidula	-15.85 to -34.29*	[4,16]	Unknown
Dictyopteris polypodioides	-16.12 to -19.8	This study,[11]	CCM
Dictyota dichotoma	-15.7 to -24.8	This study	CCM
Dictyota dichotoma var. intricata	-18.58	[11]	CCM
Dictyota spiralis	-15.06 to -24.8*	This study, [3]	CCM
Dictyota sp.	-15.06 to -24.8*	This study, [3]	CCM
Falkenbergia sp.	Unknown		Unknown
Feldmannia irregularis	-16.5*	[17]	CCM
Flabellia petiolata (*Udotea petiolata*)	-29.60 to -30.8	This study, [11]	Non-CCM
Gelidiella pannosa	-14.75*	[4]	CCM
Gelidium bipectinatum	-12.75 to 17.30*	[11,18]	CCM
Gelidium minusculum	-12.75 to 17.30*	[11,18]	CCM
Griffithsia phyllamphora	Unknown		Unknown
Halimeda tuna	-19.30	[13]	Calcareous green
Halopteris filicina	-20.10 to -22.2*	This study, [6]	CCM
Halopteris scoparia	-20.07 to -22.2	This study, [13]	CCM
Herposiphonia secunda	Unknown		Unknown
Herposiphonia secunda f. tenella	Unknown		Unknown
Herposiphonia sp.	Unknown		Unknown
Heterosiphonia crispella	-14.99 to -28.98*	[3]	Unknown
Hildenbrandia rubra	-18.04*	[3]	CCM
Hydrolithon boreale	-12.70*	[5]	Calcareous red
Hydrolithon cruciatum	-12.70*	[5]	Calcareous red
Hydrolithon farinosum	-12.70*	[5]	Calcareous red
Jania rubens	-12.57 to -23.69	[3,11]	Calcareous red
Laurencia obtusa	-9.72 to -20.26*	[3,9]	CCM
Laurencia sp.	-9.72 to -20.26*	[3,9]	CCM
Lithophyllum incrustans	Unknown		Calcareous red
Lithophyllum sp.	Unknown		Calcareous red
Lobophora variegata	-11.96 to -17.55	[3,9]	CCM
Lophosiphonia cristata	Unknown		Unknown
Meredithia microphylla	Unknown		Calcareous red
Mesophyllum sp.	Unknown		Calcareous red
Monosporus pedicellatus	Unknown		Unknown
Neogoniolithon brassica-florida	-12.40	[5]	Calcareous red
Nitophyllum punctatum	-30.55 to -33.59	[3,9]	Non-CCM
Osmundea truncata	-16.31 to -17.23*	[11,19]	CCM
Padina pavonica	-6.46 to -16.3	This study,[11]	Calcareous brown
Parvocaulis parvulum			Calcareous green
Peyssonnelia armorica	-9.77 to -32.71*	[9,20]	Calcareous red
Peyssonnelia bornetii	-9.77 to -32.71*	[9,20]	Calcareous red
Peyssonnelia cfr rubra	-9.77 to -32.71*	[9,20]	Calcareous red

Peyssonnelia dubyi	-9.77 to -32.71*	[9,20]	Calcareous red
Peyssonnelia polymorpha	-9.77 to -32.71*	[9,20]	Calcareous red
Peyssonnelia squamaria	-9.77 to -32.71*	[9,20]	Calcareous red
Phyllophora crispa	-28.73 to -34.76	[3,11]	Non-CCM
Phyllophora sicula	-28.73 to -34.76*	[3,11]	Non-CCM
Phymatolithon cfr lenormandii	Unknown		Calcareous red
Phymatolithon lenormandii	Unknown		Calcareous red
Pneophyllum fragile	Unknown		Calcareous red
Polysiphonia denudata	-14.47 to -29.39*	[3]	Unknown
Polysiphonia fibrata	-14.47 to -29.39*	[3]	Unknown
Polysiphonia scopulorum	-14.47 to -29.39*	[3]	Unknown
Pseudochlorodesmis furcellata	Unknown		Unknown
Pterocladiella capillacea	-14.50 to 20.21	[11,21]	CCM
Ptilothamnion sphaericum	Unknown		Unknown
Sargassum vulgare	-15.40 to -15.82	[3,11]	CCM
Spermothamnion repens	Unknown		Calcareous red
Spermothamnion strictum	Unknown		Calcareous red
Sphacelaria cirrosa	-17.85*	[3]	CCM
Sphacelaria rigidula	-17.85*	[3]	CCM
Sphacelaria sp.	-17.85*	[3]	CCM
Sphacelaria tribuloides	-17.85*	[3]	CCM
Titanoderma pustulatum	Unknown		Calcareous red
Titanoderma sp.	Unknown		Calcareous red
Valonia utricularis	-16.35	[11]	CCM

SI 4: Seawater carbonate chemistry and $\delta^{13}C$ of dissolved inorganic carbon (DIC) at locations from Boatta et al.[22] pH_T, A_T (total alkalinity), DIC and $\delta^{13}C$ of DIC were measured, while the species of DIC were calculated from A_T and DIC. Units are µmol kg^{-1} for all parameters except pH_T and $\delta^{13}C$ of DIC.

Site	pH_T	A_T	DIC	$[CO_2]$	$[HCO_3^-]$	$[CO_3^{2-}]$	$\delta^{13}C$ of DIC
S3	7.69 (± 0.06)	2475 (± 10)	2323 (± 29)	30 (± 7)	2164 (± 44)	128 (± 22)	-0.54 (± 0.14)
S2	7.89 (± 0.09)	2518 (± 18)	2195 (± 10)	14 (± 1)	1959 (± 25)	222 (± 18)	0.54 (± 0.04)
S1	8.04 (± 0.02)	2516 (± 11)	2170 (± 10)	12 (± 1)	1910 (± 10)	248 (± 8)	0.45 (± 0.28)

References cited in Supplementary Information:

1 Porzio, L., Buia, M. C. & Hall-Spencer, J. M. Effects of ocean acidification on macroalgal communities. *J. Exp. Mar. Biol. Ecol.* **400**, 278-287, (2011).
2 Baggini, C. *et al.* Seasonality affects macroalgal community response to increases in *p*CO_2. *PLoS. ONE* **9**, e106520, (2014).
3 Raven, J. A. *et al.* Mechanistic interpretation of carbon isotope discrimination by marine macroalgae and seagrasses. *Funct. Plant Biol.* **29**, 355-378, (2002).
4 Diaz-Pulido, G., Cornwall, C. E., Gartrell, P., Hurd, C. L. & Tran, D. V. Strategies of dissolved inorganic carbon use in macroalgae across a gradient of terrestrial influence: implications for the Great Barrier Reef in the context of ocean acidification. *Coral Reefs* **35**, DOI:10.1007/s00338-016-1481-5 (2016).
5 Wang, W.-L. & Yeh, H.-W. $\delta^{13}C$ values of marine macroalgae from Taiwan. *Bot. Bull. Acad. Sinica* **44**, 107-112, (2003).
6 Hepburn, C. D. *et al.* Diversity of carbon use strategies in a kelp forest community: implications for a high CO_2 ocean. *Glob. Change. Biol* **17**, 2488-2497, (2011).
7 Raven, J. A., Walker, D. I., Johnston, A. M., Handley, L. L. & Kübler, J. E. Implications of ^{13}C natural abundance measurements for photosynthetic performance by marine macrophytes in their natural envrionment. *Mar. Ecol. Prog. Ser.* **123**, 193-205, (1995).
8 Maberly, S. C., Raven, J. A. & Johnston, A. M. Discrimination between ^{12}C and ^{13}C by marine plants. *Oecologia* **91**, 481-492, (1992).
9 Marconi, M., Giordano, M. & Raven, J. A. Impact of taxonomy, geography, and depth on $\delta^{13}C$ and $\delta^{15}N$ variation in a large collection of macroalgae. *J. Phycol.* **47**, 1023-1035, (2011).
10 Vizzini, S. & Mazzola, A. Sources and transfer of organic matter in food webs of a Mediterranean coastal envrionment: evidence for spatial variability. *Estuar. Coast. Shelf Sci.* **66**, 459-467, (2006).
11 Mercado, J. M., de los Santos, C. B., Pérez-Lloréns, J. L. & Vergara, J. J. Carbon isotope fractionation in macroalgae from Cádiz Bay (Southern Spain): Comparison with other bio-geographic regions. *Estuar. Coast. Shelf Sci.* **85**, 449-458, (2009).
12 Raven, J. A., Johnston, A. M., Newman, J. R. & Scrimgeour, C. M. Inorganic carbon acquisition by aquatic photolithoatrophs of Dighty Burn, Angus, UK: uses and limitations of natural abundance measurements of carbon isotopes. *New Phytol.* **127**, 271-286, (1994).
13 Lepoint, G., Nyssen, F., Gobert, S., Dauby, P. & Bouquegneau, J. M. Relative impact of a seagrass bed and its adjacent epilithic algal community in consumer diets. *Marine Biology* **136**, 513-518, (2000).
14 Golléty, C., Riera, P. & Davoult, D. Complexity of food web structure of the *Ascophyllum nodosum* zone evidenced by a $\delta^{13}C$ and $\delta^{15}N$ study. *J. Sea Res.* **64**, 304-312, (2010).
15 Schaal, G., Riera, P. & Leroux, C. Trophic significance of the kelp *Laminaria digitata* (Lamour.) for the associated food web: a between-sites comparison. *Estuarine, Coastal and Shelf Science* **85**, 565-572, (2009).
16 Rigolet, C., Thiébaut, E. & Dubois, S. F. Food web structures of subtidal benthic muddy habitats: evidence of microphytobenthos contribution supported by an engineer species. *Mar. Ecol. Prog. Ser.* **500**, 25-41, (2014).

17 Falkenberg, L. J., Russell, B. D. & Connell, S. D. Contrasting resource limitations of marine primary producers: implications for competitive interactions under enriched CO2 and nutrient regimes. *Oecologia* **172**, 575-583, (2013).
18 Hill, J. M. & McQuaid, C. D. Variability in the fractionation of stable isotopes during degradation of two intertidal red algae. *Estuar. Coast. Shelf Sci.* **82**, 397-405, (2009).
19 Stepien, C. C. Impacts of geography, taxonomy and functional group on inorganic carbon use patterns in marien macrophytes. *J. Ecol.* **103**, 1372-1383, DOI:10.1111/1365-27.12451 (2015).
20 Runcie, J. W., Gurgel, C. F. D. & McDermid, K. J. *In situ* photosynthetic rates of tropical marine macroalgae at their lower depth limit. *Eur. J. Phycol.* **43**, 377-388, (2008).
21 Carvalho, M. C. & Eyre, B. D. Carbon stable isotope discrimination during respiration in three seaweed species. *Mar. Ecol. Prog. Ser.* **437**, 41-49, (2011).
22 Boatta, F. *et al.* Geochemical survey of Levante Bay, Vulcano Island (Italy), a natural laboratory for the study of ocean acidification. *Mar. Pollut. Bull.* **73**, 485-494, (2013).
